# Relationship between DXA measured systemic bone mineral density and subchondral bone cysts in postmenopausal female patients with knee osteoarthritis: a cross-sectional study: Osteoarthritis cysts and bone mineral density

**DOI:** 10.1186/s12891-023-07141-y

**Published:** 2024-01-11

**Authors:** Şükrü Burak Tönük, Zeynep Rezan Yorgancıoğlu, Selma Uysal Ramadan, Seher Kocaoğlu

**Affiliations:** 1 Department of Physical Medicine and Rehabilitation, Bolu Abant Izzet Baysal University, Karacasu, Bolu, Turkey; 2 Department of Physical Medicine and Rehabilitation, Ministry of Health Ankara Training and Research Hospital, Ankara, Turkey; 3 Department of Radiology, Ministry of Health Kecioren Training and Research Hospital, Ankara, Turkey

**Keywords:** Bone mineral density, Osteoarthritis, Subchondral bone cyst, Vitamin D

## Abstract

**Background:**

Individuals with high systemic bone mineral density (BMD) may have an increased risk of incident knee osteoarthritis (OA). Besides that, radiographic osteophytes are strongly associated with BMD. Because of these reasons, the aim of the study was to investigate the possible association between radiological subchondral bone cyst (SBC) grade and systemic BMD and vitamin D status in the postmenopausal female patients with knee OA in a crosss-sectional study.

**Methods:**

This study included of 48 osteoporosis treatment-free postmenopausal patients diagnosed with symptomatic medial compartment knee OA. BMD analysis was performed using dual-energy X-ray absorptiometry (DXA) and serum vitamin D levels were measured after recording patients’ findings. Each knee was scanned using computed tomography (CT), and categorical SBC scores were graded for the medial and lateral tibiofemoral (TF) and patellofemoral (PF) compartments and further calculated as compartmental total, total TF and grand total of both TF compartments. SBC scores were analysed with correlation analysis.

**Results:**

The patient population was characterized by radiographic joint space narrowing, obesity and low vitamin D status. Median medial total and grand total TF SBC scores were significantly different between the patient groups according to the Kellgren-Lawrence (KL) radiographic grading (*p* = 0.006 and *p* = 0.007, respectively). There were no correlations between femoral BMD values and SBC scores. However, positive correlations were detected significantly between L_1 − 4_ DXA values and TF SBC scores, but not with PF SBC scores (*p* = 0.005 for the correlation between L_1 − 4_ BMD and medial compartments total TF SBC score, *p* = 0.021 for the correlation between L_1 − 4_ BMD and grand total TF SBC score). No significant correlations were found with Vitamin D levels.

**Conclusions:**

Development of TF OA high-grade SBCs may be linked to systemic bone mass as represented by trabecular bone-rich lumbar vertebrae. The relationship might point to the importance of bone stiffness as an acting factor in knee OA possibly with mechanical energy transfer to the joint.

## Background

Osteoarthritis (OA), osteoporosis (OP) and vitamin D deficiency are among the major health problems that are frequently encountered by physicians in clinical practice. The absence of hip OA in patients with hip fracture was first documented 50 years ago [1]. The development of bone mineral density (BMD) measurement methods has provided new insights. Spinal and particularly femoral BMD values seem to be higher in patients with OA of the knee or hip than in those without OA, and this is concomitant with a increased rate of bone loss in OA progression as indicated by Kellgren-Lawrence (KL) radiographic grading [2–6]. Two cardinal radiographic features of OA, osteophytes (OST) and joint space narrowing (JSN), are associated with BMD. This association is much stronger for osteophytes than JSN suggesting that a general predisposition to hypertrophic OA subtype characterized with increased bone formation [7, 8].

Subchondral bone cysts (SBC) have been identified to be associated with cartilage loss and increased chance of joint replacement surgery in patients with OA. They induce increased intraosseous stress in OA [9–11]. They are formed by thickened trabeculae with a sclerotic wall in regions of greater mechanical stress and cartilaginous degeneration. If present, the number of cysts is more than one and their sizes are variable. Prevalent bone marrow edema-like lesions strongly predict incident SBCs in the same region [12]. The correlation between bone marrow edema and SBC size increases in the medial tibiofemoral compartment over time concomitant with the loss of cartilage volume in progressive knee OA [13].

Despite the increase in its volume fraction, subchondral bone becomes undermineralized due to the abnormal local bone remodeling with high bone turnover, which leads to a decrease in subchondral bone quality [14, 15]. The trabecular bone immediately surrounding the SBCs have a lower degree of mineralization than the contiguous trabecular bone. There may be many SBCs in severe knee OA, which are expected to play a significant role in the high local bone turnover and osteochondral changes of OA [16, 17]. However, according to a recent article, SBC number and and size may be associated with high subchondral regional BMD in both tibiofemoral compartments [18]. On the other hand, effect of systemic BMD on SBC development has not yet been fully elucidated. To our knowledge, there is only one published article in the literature investigating the association between systemic BMD values and hand SBCs using plain X-ray technique that reported a possible negative association with BMD in a Korean sample [19]. However, factors leading to the development of knee OA are different than those of hand OA. Due to the association with other radiographic cardinal features of OA, the topic needs to be elucidated with advanced research. Hence, the aim of this study was to investigate the possible association of systemic BMD and vitamin D status on SBC grading using available computed tomography (CT).

## Methods

Postmenopausal female volunteers diagnosed with knee OA in the Musculoskeletal Disorders Outpatient Clinic of Ankara Training and Research Hospital were enrolled in the study between 2002 and 2004. All patients were diagnosed with knee OA according to the American College of Rheumatology (ACR) criteria and had been suffering from knee pain on both sides and had painful knees in the last month. The study was approved by the Institutional Ethics Committee and this study was conducted in accordance with the ethical standards of the Declaration of Helsinki. Informed consent was obtained from all patients.

### Patients

The inclusion criteria were as follows: the presence of grade ≥ II symptomatic medial tibiofemoral (TF) OA in both sides according to the Kellgren-Lawrance (KL) radiographic grading system, age between 46 and 75 years, and being in the postmenopausal period. The exclusion criteria included previous or current use of vitamin D, estrogen replacement therapy or osteoporosis treatment, knee valgus/lateral TF OA [20], clinical joint effusion, previous knee surgery or trauma, inflammatory arthritis, history of knee injection, and other secondary OA and OP causes according to the patient records such as joint infection, neuropathic arthropathy, chondrocalcinosis, hypercalcemia, primary hyperparathyroidism, primary hyperthyroidism, renal disease, hepatic disease and BMD lowering drugs.

A detailed physical examination was performed. We recruited patients with painful knees judged at the time of physical examination by joint palpation and range of motion. Sex, age, occupation, disease duration, and menopause duration were recorded. A decision was made to study non-dominant extremity in order to eliminate the life-long effect of the biomechanical load of the dominant knee due to overuse. The dominant extremity was determined by the answer to the question: “If a ball is placed on the ground, with which foot would you kick the ball?” To evaluate obesity, body mass index (BMI) was calculated using the body weight/(height)^2^ formula. Height was measured in meters without shoes and body weight was measured in kilograms after removing the clothes that increase the weight.

### Pain assessment

All patients were evaluated for pain using patient-reported visual analog scale (VAS)-pain.

#### VAS-pain

The non-dominant knee pain was evaluated using the VAS during passive flexion range of motion (ROM), while the joint was flexed gently by the physician along its entire unlimited ROM when the patient was in the supine position. All patients were examined by the same physician. The VAS is a linear scale ranging from 0 to 10 cm. The left pole (0) corresponds to no pain and the right pole (10) corresponds to unbearably intense pain. The patients were asked to indicate the pain they felt during knee motion by placing a mark on the scale.

### Assessment of vitamin D level

Serum 25-hydroxyvitamin D_3_ (calcifediol) levels measured using a venous blood sample were obtained from the patients at 8^00^-12^00^ on the day following a normal dinner. The samples were evaluated with MassChrom® 25-OH-Vitamin D3/2 (ChromSystems, Munich, Germany) using a 1200 HPLC system (Agilent Technologies, Santa Clara, CA, USA ) in the hospital laboratory. Calcifediol was measured in ng/mL.

### Measurement of bone mineral density

BMD of the skeletal sites was measured by a trained X-ray technician using a GE Lunar dual-energy X-ray absorptiometry (DXA) device (Madison, WI, USA) in the regions of interest, L_1-4_ total and femoral neck. The DXA device was calibrated daily and weekly using appropriate phantom methods. Hip measurements were performed on non-dominant extremities. The measured density values were recorded in terms of the areal density (g/cm^2^), and the Z-score. The Z-score was defined as the number of standard deviations above or below the mean for the patient’s age, sex and ethnicity.

### Radiographic evaluation

In all patients, standard weight-bearing anteroposterior knee radiographs were obtained when the heels were stabilized. Body weight was evenly distributed in both extremities. The radiation dose was adjusted according to the size of the extremities and nature of the subcutaneous tissue. All radiographs were examined by the same physician and checked by another physician.

Both knees were individually evaluated based on the KL radiographic grading system. In this study, the KL grading system included three groups according to the OST and JSN grading as follows: grade II = definitive OST and/or mild JSN; grade III = mid-size OSTs and/or marked JSN; and grade IV = large, proliferative OSTs and/or advanced JSN. The classification was concluded at an advanced stage based on the OST or JSN score.

Both knees were evaluated to grade the osteophyte progression according to the KL grading system as follows: grade I = possible osteophyte lipping; grade II = definite OSTs; grade III = mid-size multiple OSTs; and grade IV = large, proliferative OSTs. Each knee was also evaluated to grade the medial tibiofemoral JSN according to the KL grading system, as follows: grade II = possible or mild JSN; grade III = marked JSN; and grade IV = advanced JSN. Subchondral sclerosis was noted as follows: 0 = absent; 1 = mild or localized eburnation; 2 = moderate increase in density; and 3 = severe, widespread sclerosis includes lateral compartment [21, 22].

### Computed tomography examination

Both knees were examined to grade SBC lesions using a Hitachi Pronto CT scanner (Chiba, Japan). During CT, the patient was placed in the supine position with the knees flexed at an angle of 30º on the table. The knees and ankles were placed adjacent to each other. In each joint, scanning was performed in the transverse plane with at slice thickness of 3 mm to include the femoral condyles and the fibular head were obtained. The coronal plane of the knee was obtained using multiplanar reconstruction. Each knee was evaluated separately for the medial and lateral TF compartments, and patellofemoral (PF) compartment. SBCs were classified into four groups as follows: 0 = none; 1 = one or two small cysts; 2 = multiple small cysts or a single large cyst; and 3 = presence of large cysts in the same compartment [22]. A total TF SBC score for the same knee that ranged between 0 and 6 was obtained by adding the SBC scores for both the medial and lateral tibiofemoral compartments. Further, medial and lateral total compartment SBC scores were separately calculated that ranged between 0 and 6 by adding the SBC scores of both dominant and non-dominant compartments of knees. Finally, the scores of all four TF compartments were added to calculate the grand total SBC score of each patient that ranged between 0 and 12. Interspinous cysts (traction cysts) were excluded as the set criteria. CT scans were evaluated by a radiologist who was blinded to the DXA measurements.

### Statistical analysis

Statistical analysises were performed using MedCalc Software version 13.0.2 (Acacialaan 22, B-8400, Ostend, Belgium). Descriptive and categorical data are expressed as the mean ± standard deviation for parametric data and the median for non-parametric data. Non-parametric variables were determined using the Shapiro-Wilk test. Differences between patient groups were evaluated using one-way ANOVA for parametric variables and the Kruskal-Wallis test for non-parametric and categorical variables. When a difference was detected between groups, a post-hoc multiple comparison test was used to determine the group from which the difference originated. The Mann-Whitney U test was used for non-parametric and categorical data to determine the group from which the difference originated. Spearman’s correlation test was used to analyze the linear relationships between the SBC scores and other variables. Statistical significance was set at P < 0.05.

## Results

A total of 48 postmenopausal women, between aged 48–75 years were included in the study. Most of the patients were immigrants from rural central Anatolia. The mean age of patients was 65.1 ± 6.7 years, mean body mass index was 31.4 ± 4.0, and mean duration of symptomatic disease was 11.7 ± 5.8 years. Recruited patients were characterized by high BMI and low vitamin D status. The descriptive statistical data are provided in Table 1 with no missing data.

**Table 1:** Demographic, clinical and radiographical findings of the patients according to the non-dominant knee Kellgren-Lawrence grading

	All patients (n:48)	K-L Grade II patients (n:17)	K-L Grade III patients (n:16)	K-L Grade IV patients (n:15)	P	Differences between the groups
Age	65.1±6.7	64.3±7.2	65.1±6.5	65.8±6.5	0.834	II = III = IV
Body mass index	31.4±4.0	30.0±4.1	31.6±2.8	32.6±4.6	0.186	II = III = IV
Calcifediol (ng/ml)	13.7 (4.3–53.1)	14.8 (4.3–42.7)	10.2 (5.2–37.8)	18.3 (5.2–53.1)	0.213	II = III = IV
L1-4 BMD	1.077±0.203	1.023±0.185	1.094±0.197	1.119±0.228	0.388	II = III = IV
L1-4 Z score	0.60±1.53	0.09±1.36	0.79±1.50	0.98±1.68	0.222	II = III = IV
Femur neck BMD	0.874±0.146	0.905±0.169	0.860±0.141	0.856±0.127	0.578	II = III = IV
Femur neck Z score	0.41±1.09	0.58±1.30	0.31±1.03	0.31±0.93	0.719	II = III = IV
VAS-pain NDK	7 (3–10)	5 (3–9)	6.5 (3–10)	7 (4–9)	0.034	II = III, II < IV**, III = IV
OST score NDK	2 (1–4)	2 (1–2)	2.5 (2–3)	3 (2–4)	0.000	II < III***, II < IV***, III < IV**
Medial JSN score NDK	3 (2–4)	2 (2–2)	3 (2–3)	4 (3–4)	0.000	II < III***, II < IV***, III < IV***
SC sclerosis score NDK	1 (0–3)	1 (0–2)	1.5 (1–3)	2 (1–3)	0.000	II < III**, II < IV***, III < IV*
NDKMCC	0.5 (0–3)	0 (0–2)	1 (0–3)	2 (0–3)	0.002	II < III*, II < IV**, III < IV*
NDKLCC	0 (0–3)	0 (0–3)	0 (0–3)	1 (0–3)	0.388	II = III = IV
NDKTC	1 (0–6)	0 (0–5)	1.5 (0–3)	3 (0–6)	0.004	II = III, II < IV**, III = IV
MCTC	2 (0–6)	1 (0–3)	2 (0–4)	3 (0–6)	0.006	II = III, II < IV**, III < IV*
LCTC	1 (0–4)	1 (0–3)	0.5 (0–4)	1 (0–4)	0.683	II = III = IV
GTC	3 (0–9)	2 (0–5)	3 (0–6)	6 (0–9)	0.007	II = III, II < IV**, III < IV*
NDKPFCC	0 (0–2)	0 (0–2)	0 (0–2)	0 (0–2)	0.775	II = III = IV

Abbreviations: NDK: non-dominant knee, NDKMCC: non-dominant knee medial compartment SBC score; NDKLCC: non-dominant knee lateral compartment SBC score; NDKTC: non-dominant knee total SBC score; MCTC: medial compartments total SBC score; LCTC: lateral compartments total SBC score; GTC: grand total SBC score of both knees, NDKPFCC: non-dominant knee patellofemoral compartment cyst score

*P < 0.05; **P < 0.01; ***P < 0.001

The patients were classified into three groups based on the non-dominant knee KL radiographic grading: grade II, 17 (35.4%) patients; grade III, 16 patients (33.3%); and grade IV, 15 patients (31.2%). During the KL grading, the JSN grade was more advanced in 22 patients, while the OST score was the determinant in 6 patients. Thus, the characterization of the patient population was closer to the atrophic OA subtype than to the hypertrophic OA subtype. There were no significant differences between the groups in terms of age, BMI, vitamin D levels, or BMD values ​​(P > 0.05).

Cysts were not detected in the non-dominant medial compartment in 24 (50.0%) patients, in the lateral compartment in 30 (62.5%) patients, and in both compartments in 17 (35.4%) patients. In both knees, medial compartment SBC scores increased significantly in parallel with an increase in the KL score (P < 0.01). No significant relationships were detected between the KL grading system and the lateral compartment SBCs (P > 0.05). The statistical results are presented in Table 1.

The details of the correlation analysis of SBCs with demographic, clinical, and radiographic findings are shown in Table 2. No correlation was detected between BMI, and serum calcifediol level (P > 0.05). Lumbar BMD values were positively correlated with all total TF SBC scores. However, the lateral compartment total SBC score did not reach significance. No correlation was detected with femur BMD values (P > 0.05). The patient-reported ipsilateral VAS pain score was correlated with non-dominant knee medial compartment SBCs in borderline significance (r: 0.283, P: 0.051), but not with non-dominant lateral SBCs (P > 0.05). Medial compartment, bi-compartmental and grand total SBC scores were positively correlated with all KL radiographic grading system features such as OST, JSN and subchondral sclerosis. In addition, PF SBC grading was neither correlated with KL radiographic grading, nor with BMD values. No correlation was observed between PF SBC grading and ipsilateral VAS-pain (P > 0.05).

**Table 2:** Correlations of subchondral cyst scores with demographic, clinical and radiographical findings

	NDKMCC (r,p)	NDKLCC (r,p)	NDKTC (r,p)	MCTC (r,p)	LCTC (r,p)	GTC (r,p)
Age	0.183 (0.213)	-0.093 (0.530)	0.051 (0.732)	0.306 (0.034)	-0.112 (0.450)	0.194 (0.187)
BMI (kg/m^**2**^)	0.085 (0.565)	0.038 (0.798)	0.098 (0.506)	0.034 (0.818)	-0.012 (0.935)	0.015 (0.920)
Calcifediol (ng/ml)	0.223 (0.128)	0.092 (0.536)	0.211 (0.150)	0.166 (0.259)	0.012 (0.934)	0.113 (0.443)
L_1 − 4_ BMD	0.298 (0.040)	0.307 (0.034)	0.353 (0.014)	0.399 (0.005)	0.165 (0.262)	0.332 (0.021)
L_1 − 4_ Z score	0.270 (0.063)	0.295 (0.042)	0.317 (0.028)	0.420 (0.003)	0.136 (0.357)	0.344 (0.017)
Femur neck BMD	-0.017 (0.908)	0.186 (0.206)	0.100 (0.499)	-0.005 (0.971)	0.148 (0.316)	0.015 (0.917)
Femur neck Z score	-0.02 (0.987)	0.136 (0.358)	0.079 (0.594)	0.067 (0.649)	0.079 (0.594)	0.048 (0.718)
OST score NDK	0.332 (0.021)	0.153 (0.301)	0.332 (0.021)	0.455 (0.001)	0.086 (0.563)	0.408 (0.004)
Medial JSN score NDK	0.507 (0.000)	0.129 (0.381)	0.459 (0.001)	0.376 (0.009)	0.006 (0.967)	0.356 (0.013)
SC sclerosis score NDK	0.389 (0.006)	0.233 (0.111)	0.415 (0.003)	0.486 (0.000)	0.105 (0.479)	0.444 (0.002)

Abbreviations: NDK: non-dominant knee, NDKMCC: non-dominant knee medial compartment SBC score; NDKLCC: non-dominant knee lateral compartment SBC score; NDKTC: non-dominant knee total SBC score; MCTC: medial compartments total SBC score; LCTC: lateral compartments total SBC score; GTC: grand total SBC score of both knees

The median SBC value was 1.0 (0–3) for the dominant knee medial compartment. SBCs were not detected in the medial compartment in 18 (37.5%) patients, in lateral compartment in 31 (64.6%) patients, and in both compartments in 11 (22.9%) patients. The dominant medial compartment SBC score was also correlated with the L_1-4_ BMD and Z score (r: 0.284, P: 0.050, and r: 0.368, P: 0.010, respectively). It was more strongly correlated with ipsilateral VAS-pain than the non-dominant medial compartment SBC score (r: 0.434, P: 0.002).

## Discussion

The patients in this study were characterized by a high body mass index, prominent JSN progression and a low vitamin D status. Medial compartment SBCs were positively correlated with all radiographic features. As expected, the medial compartment SBC scores were higher in most patients in parallel with the KL grading. However, we observed that there was no significant difference between the KL patient groups in terms of the grading of lateral compartment SBCs. Most SBC scores were significantly correlated with trabecular bone-rich lumbar BMD scores, as strongest for the medial compartments total SBC score. No significant correlations were detected with vitamin D level. This is the first work to evaluate the association of systemic BMD with knee OA SBCs.

Increased SBC number and volume as determined by quantitative CT (QCT), were reported in a recently published study to be correlated with high regional tibia BMD in both the medial and lateral compartments but not with BMD of the total proximal tibia [18]. The authors of that study have concluded that it might reflect tissue resistance around the cysts to withstand against to the mechanical load. According to the same study, only lateral SBCs were correlated with knee malalignment, lateral KL grading, and lateral JSN. The study population consisted of patients including valgus OA, and the patient phenotype may be more close to hypertrophic OA than atrophic OA.

The pathogenesis of OA cysts has not yet been fully elucidated. SBCs are generally observed in areas with articular cartilage degeneration and they are generally communicate to the joint space through a defect in the bone. Many investigators argue that cysts are formed as a result of the passage of synovial fluid to the subchondral bone marrow through defects (cracks) existing both in the degenerated articular cartilage and on the cortical bone surface due to increased intra-articular pressure (hydrodynamic theory) or as a result of osteonecrosis caused by excessive mechanical stress (osseous contusion theory) [23, 24]. Some authors have suggested that the strong correlation of SBCs with bone marrow edema-like lesions in the same region supports the osseous contusion theory [12]. It is likely that high intra-articular pressure, low grade local inflammation, excessive mechanical stress, and low-quality of subchondral bone cause the joint fluid to pass to the trabecular area by forming a defect on the bone surface, thereby, triggering SBC formation. Cyst formation leads to an impaired blood circulation and the development of bone necrosis resulting in stress fractures around the cyst [25]. As cysts are generally formed just below the articular surface, collapse of the surface into the cyst cavity is commonly seen but is a late-occurring phenomenon. A histological analysis showed that non-communicating SBCs contain necrotic bone fragments with dead denuclearized cells [26]. This fact might be related to the possible regression of a SBC as a result of improvement of the bone defect, or might be considered as a clue for osseous contusion.

In this study, lumbar BMD values were correlated with most of the SBC scores, especially with medial compartments total SBC and grand total SBC, but not femoral BMD. Although it seems plausible, we could not detect a linear relationship of SBCs with BMI as pointed also by previous studies [9, 18], despite the fact that a considerable number of study patients presented with genu varum deformity. As another biomechanical standpoint apart from the joint compression due to body weight, bone stiffness might have an additive causative role in the condition of high systemic BMD. Structural stiffness is a measure to resistance to deformation under the applied mechanical load with high strength, but less elasticity. On the other hand, correlation between stiffness and strength attenuates with aging [27–29]. Bone’s shock absorption feature is decreased due to the increased stiffness because of less energy dissipation. Mechanical force vectors like ground reaction force (GRF) that is transmitted through tibia during the stance phase of the gait cycle (heel strike at the stabile position of knee in full extension), might cause additional micro-damage (cracks) in the knee structures in a time-dependent manner, together with enhancing the bone marrow edema-like lesions in the regional trabecular bone [30]. Besides that, small cysts without cartilage lesions and bone marrow edema are more common in highly active-trained runners that seems to be not compatible with hydrodynamic theory [30]. Therefore, patient-specific gait pattern should also be considered in the pre-OA phase. However, systemic BMD loss accelerates with radiographic advancement of knee OA as indicated by DXA and speed of sound (SOS) based on quantitative ultrasound imaging (QUS) [3, 6, 31]. The peak adduction moment increases in patients with OA, whereas the frontal GRF decreases as adaptive gait changes possibly due to the medial JSN [32].

Vitamin D levels were not correlated with the SBC grading, although vitamin D receptor gene polymorphisms might be of importance [33]. Nevertheless, we think that some cellular and molecular aspects of vitamin D should be discussed. Non-epithelial fibrous walls of OA SBCs have variable thickness and are commonly surrounded by bone trabeculae that show alterations consistent with increased osteoblastic and osteoclastic activity [25]. There is a chronic inflammatory infiltration especially rich in macrophages in both the walls and lumen of SBCs. Fibrous tissue of the cystic wall is regarded to actively release inflammatory mediators, and cytokines such as interleukin (IL)-1, IL-6, and tumor necrosis factor (TNF)-α [34]. These cytokines account for the cystic enlargement. Macrophage-derived osteoclastic resorption plays an important role in subchondral bone remodeling [35]. Osteoarthritis SBCs contain numerous CD 68(+) macrophages. These cells are also abundant in the edematous and fibrotic bone marrow surrounding the cyst. Macrophages isolated from the walls of OA SBCs can be transformed into osteoclasts with the lacunar resorption ability in vitro [35]. However, osteoblastic cells and calcitriol are essential for in vitro macrophage transformation. The macrophages obtained from OA cysts do not need the existence of macrophage colony stimulating factor (M-CSF). Due to this property, they differ from the macrophages obtained from the synovial fluid of the patients with rheumatoid arthritis (RA) [36]. A prominent consideration is the requirement of calcitriol and osteoblastic cells in the environment for the transformation from macrophages to osteoclasts in OA cysts [35]. Calcitriol synthesized from calcifediol by macrophages may play an important role in this process [37–39].

Calcitriol can be synthesized in extra-renal tissues that are not under homeostatic control, and its production is substrate-dependent [39, 40]. Therefore, locally produced calcitriol may promote the enlargement of SBCs owing to its paracrine effects. If this condition is true, severe vitamin D deficiency might slow aberrant cyst enlargement in OA. A similar pathophysiological mechanism depended on calcitriol might also be involved in cartilage degradation [41, 42]. On the other hand, vitamin D deficiency may adversely effects bone quality as endocrine, and promotes the proximal medial collapse of tibia.

In summary, the main event that promotes the development of symptomatic knee OA might be the different subchondral bone behavior driven by systemic bone mass with certain conditions. Progression process is represented also by the disordered subchondral bone, which may be vulnerable to biomechanical factors just as the cartilage. It is clear that obesity, bone mass, and lifestyle have a first-degree role in the pathophysiology of knee OA. However, other possible acting factors such as tissue stiffness and differences in neural mechanisms of locomotion should also be considered. It is worth noting that less stiff subchondral bone may be prone to repetitive micro-damage by absorbing more mechanical energy, and thereby spares the overlying cartilage. However, stiff bone with the decreased ability of energy absorption may cause to repetitive micro-damage of articular cartilage [43]. Furthermore, the SBCs may be associated mainly with female gender and development of varus deformity [44]. Therefore, involvement of bone stiffnes in the development of SBCs needs to be elucidated by considering the deformities of knee as determined by the femoral and tibial joint angle measurements.

This study has several limitations. Three-dimensional CT or magnetic resonance imaging (MRI) may have been a better method to determine the SBC status. However, two-dimensional CT images can reveal the occurrence of osseous macro-cardinal features of OA better than radiography, correlated with MRI [22]. Secondly, the recruited female patient sample size was small. Finally, most volunteers had a high BMI and low vitamin D status. Many patients could not be enrolled in the study due to a history of estrogen replacement therapy, osteoporosis treatment or vitamin D supplementation. However, this condition was a major strength of this study when the life-long effects of vitamin D are needed to investigate. Thus, the findings should not be generalized to male OA and patients with low BMI.

## Conclusion

Positive correlations were detected in postmenopausal female OA patients between trabecular bone rich lumbar BMD and TF SBC grading, but not with PF SBCs. Statistical analyses have showed that the relationships were slightly stronger in the medial TF compartment SBC grade as it increases with radiographic progression, but low-grade lateral TF compartment SBCs were not uncommon in the study patients. Further studies are needed to elucidate the importance of SBCs in the pathophysiology of OA with the consideration of BMD status in different relevant aspects.

## Data Availability

The analyzed dataset of this study is available from the corresponding author on reasonable request.

## Abbreviations

BMD: Bone mineral density
BMI: Body mass index
CD: Cluster of differentiation
CT: Computed tomography
DXA: Dual-energy X-ray absorptiometry
GRF: Ground reaction force
IL: Interleukin
JSN: Joint space narrowing
KL: Kellgren-Lawrence
M-CSF: Macrophage colony stimulating factor
MRI: Magnetic resonance imaging
OA: Osteoarthritis
OP: Osteoporosis
OST: Osteophytes
PF: Patellofemoral
QUS: Quantitative ultrasound
RA: Rheumatoid arthritis
ROM: Range of motion
SBC: Subchondral bone cyst
SOS: Speed of sound
TF: Tibiofemoral
TNF: Tumor necrosis factor
VAS: Visual analog scale
GTC: Grand total SBC score of both knees
LCTC: Lateral compartments total SBC score
MCTC: Medial compartments total SBC score
NDK: Non-dominant knee
NDKLCC: Non-dominant knee lateral compartment SBC score
NDKMCC: Non-dominant knee medial compartment SBC score
NDKPFCC: Non-dominant knee patellofemoral compartment cyst score
NDKTC: Non-dominant knee total SBC score
